# Bis[4-chloro-2-(quinolin-8-yl­imino­meth­yl)phenolato-κ^3^ *N*,*N*′,*O*]cobalt(III) trichlorido­methano­lcobaltate(II)

**DOI:** 10.1107/S1600536813010118

**Published:** 2013-04-20

**Authors:** Xu-Jian Luo, Chuan-Hui Zhang, Jie Zhou, Yan-Cheng Liu

**Affiliations:** a College of Chemistry and Chemical Engineering, Central South University, Changsha 410083, People’s Republic of China; b School of Chemistry & Chemical Engineering of Guangxi Normal University, Guilin 541004, People’s Republic of China

## Abstract

The reaction of 4-chloro-2-(quinolin-8-yl­imino­meth­yl)phenol (HClQP) with cobalt(II) dichloride hexa­hydrate in methanol/chloro­form under solvothermal conditions yielded the title compound, [Co(C_16_H_10_ClN_2_O)_2_][CoCl_3_(CH_3_OH)]. The Co^III^ atom is six-coordinated in a slightly distorted octa­hedral geometry by four N atoms and two O atoms of two tridentate HClQP ligands, which are nearly perpendicular to each other, making a dihedral angle of 86.95°. The Co^II^ atom is four-coordinated by three Cl atoms and one O atom from a methanol ligand in a distorted tetra­hedral geometry. The crystal packing is consolidated by inter­molecular O—H⋯Cl, C—H⋯Cl and C—H⋯O hydrogen bonds, forming a three-dimensional supra­molecular structure, in which [Co^II^Cl_3_(CH_3_OH)] anions are connected *via* O—H⋯Cl and C—H⋯Cl hydrogen bonds into centrosymmetric dimers. Neighboring cobalt(III) complexes form dimers through C—H⋯O hydrogen bonds, as well as π–π stacking [centroid–centroid distances = 3.30 (2) Å] between the planar quinoline systems of one HClQP ligand and the phenolate ring of another.

## Related literature

For the synthesis and analysis of the HClQP ligand, see: Donia & El-Boraey (1993 ▶), Sirirak *et al.* (2013 ▶). For related crystal structures of metal complexes of HClQP, see: Vasil’chenko *et al.* (1999 ▶); Neves *et al.* (2009 ▶). For applications of metal complexes of Schiff bases and their biological activity, catalytic reactions and photoelectric properties, see: Wu *et al.* (2009 ▶); Zhuang *et al.* (2010 ▶); Leung *et al.* (2011 ▶).
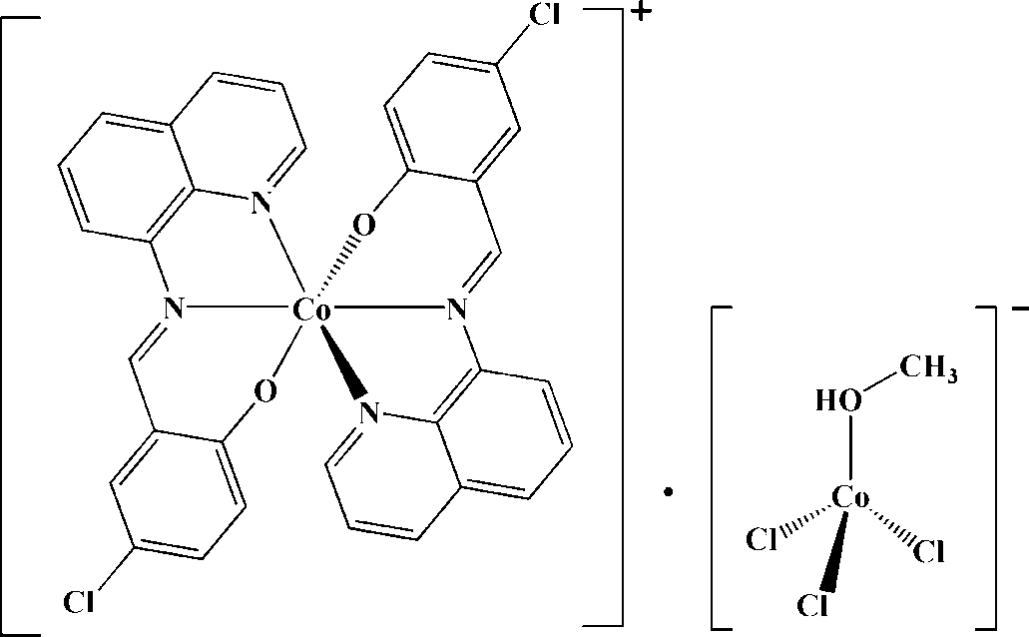


## Experimental

### 

#### Crystal data

[Co(C_16_H_10_ClN_2_O)_2_][CoCl_3_(CH_4_O)]
*M* *_r_* = 819.67
Triclinic, 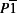

*a* = 12.0547 (6) Å
*b* = 12.1822 (4) Å
*c* = 13.2435 (7) Å
α = 65.156 (4)°
β = 83.108 (4)°
γ = 68.444 (4)°
*V* = 1640.06 (13) Å^3^
*Z* = 2
Mo *K*α radiation
μ = 1.46 mm^−1^
*T* = 293 K
0.40 × 0.20 × 0.12 mm

#### Data collection

Agilent SuperNova diffractometer
Absorption correction: multi-scan (*CrysAlis PRO*; Agilent, 2011 ▶) *T*_min_ = 0.809, *T*_max_ = 1.000
14351 measured reflections
6694 independent reflections
5581 reflections with *I* > 2σ(*I*)
*R*_int_ = 0.025

#### Refinement

*R*[*F* ^2^ > 2σ(*F* ^2^)] = 0.035
*wR*(*F* ^2^) = 0.091
*S* = 1.05
6694 reflections
428 parameters
2 restraints
H atoms treated by a mixture of independent and constrained refinement
Δρ_max_ = 0.42 e Å^−3^
Δρ_min_ = −0.38 e Å^−3^

### 

Data collection: *CrysAlis PRO* (Agilent, 2011 ▶); cell refinement: *CrysAlis PRO*; data reduction: *CrysAlis PRO*; program(s) used to solve structure: *SHELXS97* (Sheldrick, 2008 ▶); program(s) used to refine structure: *SHELXL97* (Sheldrick, 2008 ▶); molecular graphics: *OLEX2* (Dolomanov *et al.*, 2009 ▶); software used to prepare material for publication: *OLEX2*.

## Supplementary Material

Crystal structure: contains datablock(s) I, global. DOI: 10.1107/S1600536813010118/zl2537sup1.cif

Structure factors: contains datablock(s) I. DOI: 10.1107/S1600536813010118/zl2537Isup2.hkl

Additional supplementary materials:  crystallographic information; 3D view; checkCIF report

## Figures and Tables

**Table 1:** Hydrogen-bond geometry (Å, °)

*D*—H⋯*A*	*D*—H	H⋯*A*	*D*⋯*A*	*D*—H⋯*A*
O3—H3*A*⋯Cl4^i^	0.85 (2)	2.25 (2)	3.081 (3)	166 (2)
C1—H1⋯Cl3^ii^	0.93	2.72	3.532 (3)	146
C10—H10⋯O1^iii^	0.93	2.47	3.324 (3)	152
C33—H33*A*⋯Cl5^i^	0.96	2.82	3.745 (4)	161

Symmetry codes: (i) 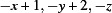; (ii) 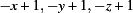; (iii) 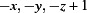.

**Fig. 1.**
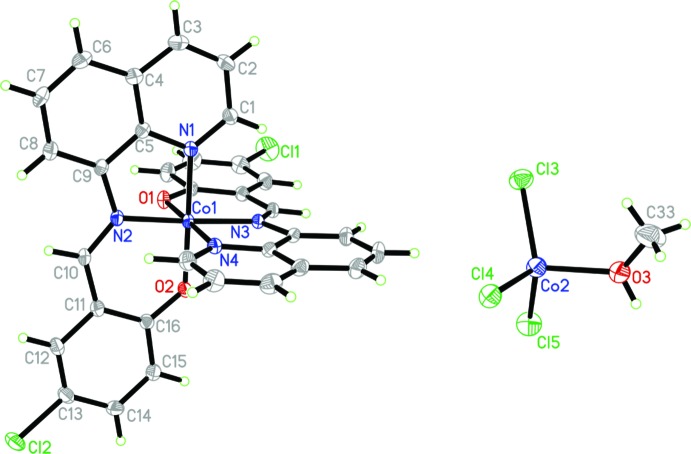
The molecular structure showing the atom-labelling scheme. Displacement ellipsoids are drawn at the 30% probability level.

**Fig. 2.**
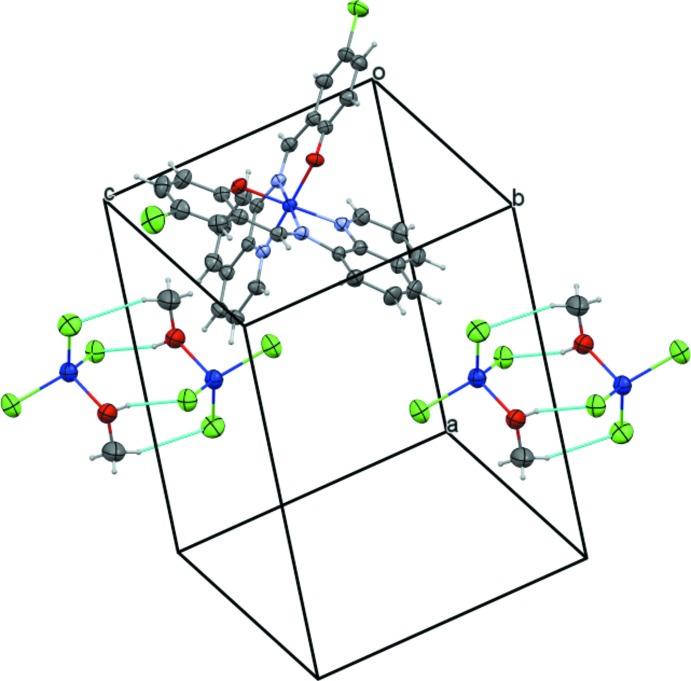
Dimeric presentations of the anionic complexes connected by C–H···Cl and O–H···Cl hydrogen bonding.

**Fig. 3.**
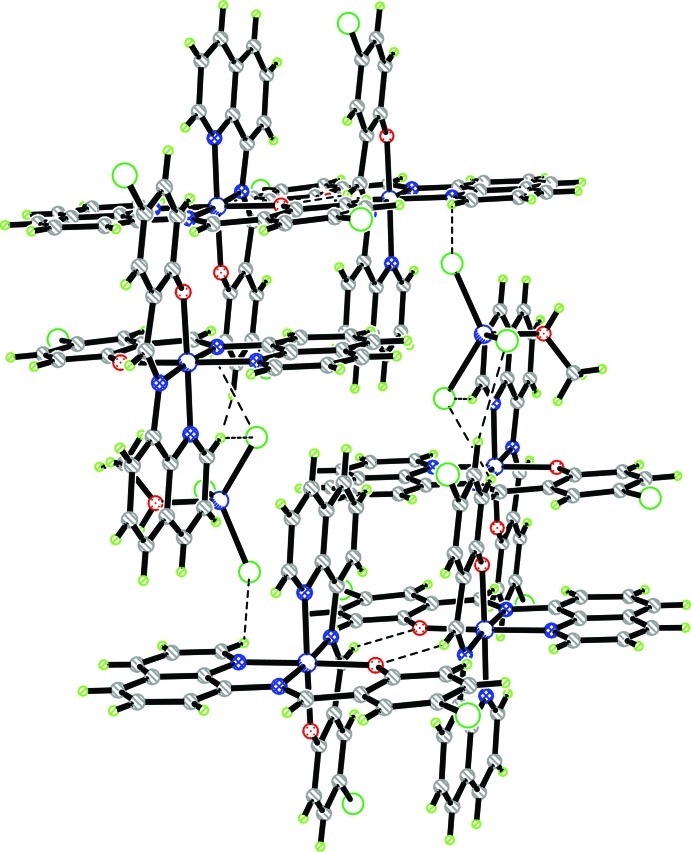
The neighboring two cationic complexes showing the π-π stacking interaction between the aromatic rings of the ClQP ligands.

**Fig. 4.**
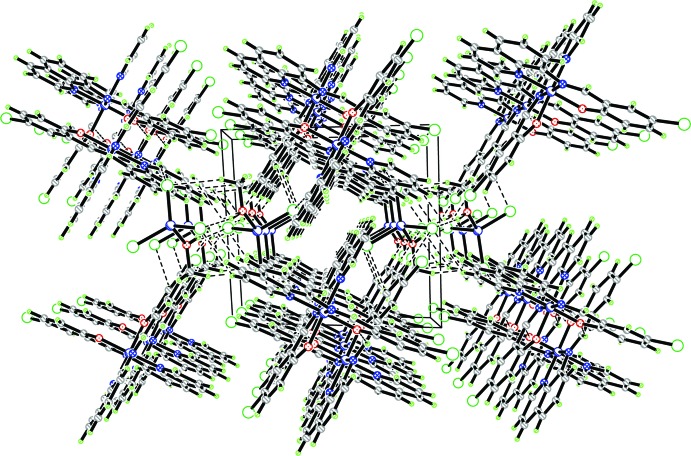
Packing diagram viewed along the b-axis.

## supplementary crystallographic information

### Comment

Coordination chemistry research on metal complexes of Schiff bases has shown their potential for applications based on their biological activities, catalytic reactions and as photoelectric materials. 4-Chloro-2-(quinolin-8-yliminomethyl)-phenol (HClQP) is a Schiff base synthesized by condesation of 8-aminoquinoline and 5-chloro-2-hydroxybenzaldehyde. It can be used as an *N*,*N*,*O*–tridentate ligand for the synthesis of new metal complexes of Schiff bases. However, few crystal structural studies on the metal complexes of HClQP have so far been reported (Neves *et al.*, 2009; Vasil'chenko *et al.*, 1999). In the present work, we report the first cobalt(III) complex of HClQP in cationic form, with a simple cobalt(II) complex in anionic form as the counterion (Fig. 1).

Crystal structure refinement of the title complex revealed that half of the cobalt(II) of the starting material was oxidized to cobalt(III) under the solvothermal synthesis conditions. The cobalt(III) is six-coordinated by four N atoms and two O atoms of two HClQP ligands, both of which are in the same tridentate coordination mode *via* the deprotonated phenol O atoms, the quinoline N atoms and the N atoms from the Schiff base C=N, respectively, to form a slightly distorted octahedron. In each of the two coordinating ClQPs, two N atoms from the respective Schiff base C=N occupy the *trans* position of the octahedron (N2—Co1—N3 176.22 (8)°), while the two phenol O atoms and quinoline N atoms occupy the *cis* positions of the octahedron (O1—Co1—O2 90.31 (7)°, N1—Co1—N4 92.24 (8)°). As a result the two tridentate ClQPs are nearly perpendicular to each other, with a dihedral angle of 91.2°. The unoxidized cobalt(II) ion, on the other hand, is four-coordinated respectively by three Cl atoms and one O atom from a methanol solvate molecule, to form a distorted tetrahedron (Fig. 1).

The crystal packing is consolidated by intermolecular O–H···Cl, C–H···Cl and C–H···O hydrogen bonds to form a supramolecular structure, (Fig. 2). Each two [Co^II^Cl_3_(CH_3_OH)] anions are connected *via* O–H···Cl and C–H···Cl hydrogen bonds into centrosymmetric dimers, in which the chlorides are all from [Co^II^Cl_3_(CH_3_OH)]^-^, and the 4-Cl atom on the phenol group of HClQP ligand does not participate in the formation of the hydrogen bonding. Neighboring cobalt(III) complexes form dimers through C–H···O hydrogen bonds, as well as *π*-*π* stacking between the planar quinoline of one HClQP ligand and the phenol ring of another.

### Experimental

Preparation of 4-Chloro-2-(quinolin-8-yliminomethyl)-phenol (HClQP):

A mixture of 5-chloro-2-hydroxybenzaldehyde (1 mmol, 0.156 g) and 8-aminoquinoline (1 mmol, 0.144 g) in anhydrous ethanol (30 ml) was stirred for 3 h at 323 K and then was concentrated to *ca* 10 ml. Hexane was then added to the solution and it was allowed to cool down to room temperature. The orange precipitate that formed was filtered off, washed with ethanol and then dried in vacuum at room temperature (yield: 0.216 g, 0.76 mmol, 76%). The analysis data (including IR, UV-vis) for HClQP are identical to those given in the original literature Donia *et al.* (1993), Sirirak *et al.* (2013).

Synthesis of the title complex:

CoCl_2_.6H_2_O (0.1 mmol, 0.023 g), HClQP (0.2 mmol, 0.056 g), 0.5 ml of ethanol and 0.3 ml of chloroform were placed in a thick Pyrex tube. The mixture was frozen by liquid N_2_, evacuated under vacuum and sealed. The mixture in the tube was then reacted at 363 K for 2 days. Dark green block crystals suitable for X-ray single-crystal diffraction analysis were harvested (yield: 0.060 g, 0.073 mmol, 73%). m.p. > 573 K (decomposed). IR (KBr, cm^-1^): 3434 (*b*), 2972 (w), 2906 (w), 1580 (*m*), 1563 (*s*), 1505 (*s*), 1487 (*s*), 1386 (*s*), 1274 (*s*), 1108 (*s*), 1090 (*s*), 1034 (*s*), 624 (*s*). UV-vis (tris buffer solution containing 1% of DMSO): ε_227 nm_ = 6.76×10^4^, ε_264 nm_ = 4.12×10^4^, ε_227 nm_ = 1.40×10^4^ (*L*×mol^-1^×cm^-1^).

### Refinement

C-bound H atoms were geometrically positioned (C—H 0.93 Å) and allowed to ride on their parent atoms, with *U*_iso_(H) = 1.2 *U*_eq_(C). N– and O– bound H atoms were located in a difference map and refined isotropically with restraints (O—H = 0.85 (2) Å; N—H = 0.90 (2) Å).

### Crystal data

**Table:**

[Co(C_16_H_10_ClN_2_O)_2_][CoCl_3_(CH_4_O)]	*Z* = 2
*M**_r_* = 819.67	*F*(000) = 826
Triclinic, *P*1	*D*_x_ = 1.660 Mg m^−^^3^
*a* = 12.0547 (6) Å	Mo *K*α radiation, λ = 0.7107 Å
*b* = 12.1822 (4) Å	Cell parameters from 6497 reflections
*c* = 13.2435 (7) Å	θ = 3.1–28.7°
α = 65.156 (4)°	µ = 1.46 mm^−^^1^
β = 83.108 (4)°	*T* = 293 K
γ = 68.444 (4)°	Block, red
*V* = 1640.06 (13) Å^3^	0.40 × 0.20 × 0.12 mm

### Data collection

**Table:**

Agilent SuperNova diffractometer	6694 independent reflections
Radiation source: SuperNova (Mo) X-ray Source	5581 reflections with *I* > 2σ(*I*)
Mirror monochromator	*R*_int_ = 0.025
Detector resolution: 16.1623 pixels mm^-1^	θ_max_ = 26.4°, θ_min_ = 3.1°
ω scans	*h* = −12→15
Absorption correction: multi-scan (*CrysAlis PRO*; Agilent, 2011)	*k* = −15→15
*T*_min_ = 0.809, *T*_max_ = 1.000	*l* = −16→16
14351 measured reflections	

### Refinement

**Table:**

Refinement on *F*^2^	Primary atom site location: structure-invariant direct methods
Least-squares matrix: full	Secondary atom site location: difference Fourier map
*R*[*F*^2^ > 2σ(*F*^2^)] = 0.035	Hydrogen site location: inferred from neighbouring sites
*wR*(*F*^2^) = 0.091	H atoms treated by a mixture of independent and constrained refinement
*S* = 1.05	*w* = 1/[σ^2^(*F*_o_^2^) + (0.038*P*)^2^ + 0.697*P*] where *P* = (*F*_o_^2^ + 2*F*_c_^2^)/3
6694 reflections	(Δ/σ)_max_ = 0.002
428 parameters	Δρ_max_ = 0.42 e Å^−^^3^
2 restraints	Δρ_min_ = −0.38 e Å^−^^3^

### Special details

**Table:**

Geometry. All e.s.d.'s (except the e.s.d. in the dihedral angle between two l.s. planes) are estimated using the full covariance matrix. The cell e.s.d.'s are taken into account individually in the estimation of e.s.d.'s in distances, angles and torsion angles; correlations between e.s.d.'s in cell parameters are only used when they are defined by crystal symmetry. An approximate (isotropic) treatment of cell e.s.d.'s is used for estimating e.s.d.'s involving l.s. planes.
Refinement. Refinement of *F*^2^ against ALL reflections. The weighted *R*-factor *wR* and goodness of fit *S* are based on *F*^2^, conventional *R*-factors *R* are based on *F*, with *F* set to zero for negative *F*^2^. The threshold expression of *F*^2^ > σ(*F*^2^) is used only for calculating *R*-factors(gt) *etc*. and is not relevant to the choice of reflections for refinement. *R*-factors based on *F*^2^ are statistically about twice as large as those based on *F*, and *R*- factors based on ALL data will be even larger.

### Fractional atomic coordinates and isotropic or equivalent isotropic displacement parameters (Å^2^)

**Table:**

	*x*	*y*	*z*	*U*_iso_*/*U*_eq_	
Co1	0.12319 (3)	0.22080 (3)	0.45839 (3)	0.02213 (9)	
Co2	0.48753 (3)	0.83377 (4)	0.18040 (3)	0.04005 (11)	
Cl1	−0.04188 (9)	0.30890 (10)	0.96844 (7)	0.0698 (3)	
Cl2	−0.34542 (7)	0.29977 (8)	0.11636 (6)	0.0517 (2)	
Cl3	0.58421 (7)	0.67622 (7)	0.33927 (6)	0.0530 (2)	
Cl4	0.50140 (7)	0.75973 (7)	0.04692 (6)	0.04683 (18)	
Cl5	0.30291 (7)	0.96359 (8)	0.19355 (7)	0.0586 (2)	
O1	0.04598 (15)	0.15626 (15)	0.59254 (14)	0.0292 (4)	
O2	−0.02554 (14)	0.34325 (15)	0.38741 (14)	0.0290 (4)	
O3	0.57844 (19)	0.9576 (2)	0.12593 (19)	0.0539 (5)	
H3A	0.5441 (19)	1.0347 (16)	0.079 (2)	0.081*	
N1	0.27402 (16)	0.09373 (18)	0.53280 (16)	0.0249 (4)	
N2	0.11356 (16)	0.08503 (17)	0.42660 (16)	0.0243 (4)	
N3	0.14397 (16)	0.35254 (17)	0.48906 (16)	0.0246 (4)	
N4	0.19894 (17)	0.28896 (18)	0.32078 (16)	0.0253 (4)	
C1	0.3534 (2)	0.1060 (2)	0.5853 (2)	0.0313 (5)	
H1	0.3399	0.1862	0.5852	0.038*	
C2	0.4562 (2)	0.0025 (3)	0.6404 (2)	0.0373 (6)	
H2	0.5100	0.0144	0.6760	0.045*	
C3	0.4772 (2)	−0.1157 (3)	0.6418 (2)	0.0380 (6)	
H3	0.5443	−0.1854	0.6802	0.046*	
C4	0.3971 (2)	−0.1327 (2)	0.5848 (2)	0.0309 (5)	
C5	0.2954 (2)	−0.0244 (2)	0.53189 (19)	0.0251 (5)	
C6	0.4131 (2)	−0.2495 (2)	0.5768 (2)	0.0391 (6)	
H6	0.4798	−0.3224	0.6108	0.047*	
C7	0.3306 (2)	−0.2552 (2)	0.5191 (2)	0.0388 (6)	
H7	0.3426	−0.3324	0.5137	0.047*	
C8	0.2285 (2)	−0.1485 (2)	0.4681 (2)	0.0325 (6)	
H8	0.1729	−0.1555	0.4304	0.039*	
C9	0.2103 (2)	−0.0326 (2)	0.47373 (19)	0.0246 (5)	
C10	0.0272 (2)	0.0961 (2)	0.3685 (2)	0.0272 (5)	
H10	0.0291	0.0219	0.3628	0.033*	
C11	−0.0693 (2)	0.2116 (2)	0.3132 (2)	0.0275 (5)	
C12	−0.1502 (2)	0.2056 (2)	0.2477 (2)	0.0326 (6)	
H12	−0.1372	0.1285	0.2417	0.039*	
C13	−0.2457 (2)	0.3108 (3)	0.1938 (2)	0.0348 (6)	
C14	−0.2673 (2)	0.4280 (3)	0.2014 (2)	0.0394 (6)	
H14	−0.3329	0.5002	0.1634	0.047*	
C15	−0.1912 (2)	0.4358 (2)	0.2651 (2)	0.0349 (6)	
H15	−0.2064	0.5141	0.2697	0.042*	
C16	−0.0908 (2)	0.3290 (2)	0.3237 (2)	0.0266 (5)	
C17	0.2202 (2)	0.2537 (3)	0.2361 (2)	0.0340 (6)	
H17	0.1974	0.1868	0.2404	0.041*	
C18	0.2758 (3)	0.3136 (3)	0.1404 (2)	0.0416 (6)	
H18	0.2880	0.2871	0.0823	0.050*	
C19	0.3119 (3)	0.4100 (3)	0.1323 (2)	0.0428 (7)	
H19	0.3503	0.4486	0.0695	0.051*	
C20	0.2906 (2)	0.4514 (2)	0.2206 (2)	0.0343 (6)	
C21	0.2326 (2)	0.3884 (2)	0.3135 (2)	0.0281 (5)	
C22	0.2056 (2)	0.4247 (2)	0.4045 (2)	0.0268 (5)	
C23	0.2403 (2)	0.5206 (2)	0.4037 (2)	0.0373 (6)	
H23	0.2244	0.5447	0.4634	0.045*	
C24	0.3000 (3)	0.5822 (3)	0.3119 (3)	0.0474 (7)	
H24	0.3235	0.6467	0.3122	0.057*	
C25	0.3246 (3)	0.5502 (3)	0.2223 (3)	0.0452 (7)	
H25	0.3636	0.5932	0.1625	0.054*	
C26	0.1089 (2)	0.3756 (2)	0.5764 (2)	0.0286 (5)	
H26	0.1202	0.4455	0.5797	0.034*	
C27	0.0542 (2)	0.3028 (2)	0.6683 (2)	0.0285 (5)	
C28	0.0293 (2)	0.3377 (3)	0.7608 (2)	0.0365 (6)	
H28	0.0457	0.4078	0.7577	0.044*	
C29	−0.0180 (3)	0.2699 (3)	0.8533 (2)	0.0430 (7)	
C30	−0.0474 (3)	0.1671 (3)	0.8581 (2)	0.0465 (7)	
H30	−0.0815	0.1223	0.9212	0.056*	
C31	−0.0260 (2)	0.1325 (3)	0.7698 (2)	0.0388 (6)	
H31	−0.0470	0.0645	0.7738	0.047*	
C32	0.0270 (2)	0.1967 (2)	0.6726 (2)	0.0273 (5)	
C33	0.7040 (3)	0.9195 (4)	0.1170 (3)	0.0818 (12)	
H33A	0.7233	0.9406	0.0402	0.123*	
H33B	0.7399	0.8279	0.1590	0.123*	
H33C	0.7341	0.9640	0.1457	0.123*	

### Atomic displacement parameters (Å^2^)

**Table:**

	*U*^11^	*U*^22^	*U*^33^	*U*^12^	*U*^13^	*U*^23^
Co1	0.02356 (17)	0.02188 (16)	0.02563 (18)	−0.01028 (13)	0.00196 (12)	−0.01228 (14)
Co2	0.0457 (2)	0.0405 (2)	0.0369 (2)	−0.01543 (18)	0.00250 (17)	−0.01841 (18)
Cl1	0.0877 (7)	0.0921 (7)	0.0468 (5)	−0.0313 (5)	0.0179 (4)	−0.0483 (5)
Cl2	0.0524 (5)	0.0598 (5)	0.0465 (4)	−0.0271 (4)	−0.0179 (3)	−0.0136 (4)
Cl3	0.0655 (5)	0.0454 (4)	0.0424 (4)	−0.0123 (4)	−0.0077 (4)	−0.0163 (4)
Cl4	0.0603 (5)	0.0453 (4)	0.0401 (4)	−0.0191 (3)	0.0048 (3)	−0.0225 (3)
Cl5	0.0522 (5)	0.0614 (5)	0.0609 (5)	−0.0119 (4)	0.0104 (4)	−0.0328 (4)
O1	0.0342 (9)	0.0306 (9)	0.0320 (10)	−0.0185 (7)	0.0073 (7)	−0.0165 (8)
O2	0.0271 (9)	0.0270 (8)	0.0373 (10)	−0.0084 (7)	−0.0038 (7)	−0.0168 (8)
O3	0.0486 (13)	0.0491 (12)	0.0562 (15)	−0.0208 (10)	−0.0044 (10)	−0.0093 (11)
N1	0.0255 (10)	0.0246 (10)	0.0273 (11)	−0.0108 (8)	0.0022 (8)	−0.0115 (9)
N2	0.0259 (10)	0.0226 (9)	0.0282 (11)	−0.0105 (8)	0.0019 (8)	−0.0125 (9)
N3	0.0246 (10)	0.0209 (9)	0.0299 (11)	−0.0087 (8)	−0.0015 (8)	−0.0104 (9)
N4	0.0263 (10)	0.0254 (10)	0.0260 (11)	−0.0099 (8)	0.0012 (8)	−0.0115 (9)
C1	0.0313 (13)	0.0324 (13)	0.0340 (14)	−0.0123 (11)	−0.0008 (10)	−0.0156 (11)
C2	0.0324 (14)	0.0446 (15)	0.0367 (15)	−0.0123 (12)	−0.0067 (11)	−0.0173 (13)
C3	0.0307 (14)	0.0388 (15)	0.0337 (15)	−0.0067 (11)	−0.0043 (11)	−0.0082 (12)
C4	0.0297 (13)	0.0315 (13)	0.0292 (14)	−0.0104 (10)	0.0034 (10)	−0.0111 (11)
C5	0.0240 (12)	0.0243 (11)	0.0264 (12)	−0.0098 (9)	0.0055 (9)	−0.0098 (10)
C6	0.0363 (15)	0.0274 (13)	0.0435 (17)	−0.0036 (11)	−0.0001 (12)	−0.0111 (12)
C7	0.0464 (16)	0.0236 (12)	0.0468 (17)	−0.0099 (11)	0.0050 (13)	−0.0176 (12)
C8	0.0383 (14)	0.0286 (13)	0.0354 (15)	−0.0140 (11)	0.0032 (11)	−0.0162 (11)
C9	0.0267 (12)	0.0236 (11)	0.0252 (12)	−0.0115 (10)	0.0059 (9)	−0.0105 (10)
C10	0.0300 (13)	0.0267 (12)	0.0329 (14)	−0.0136 (10)	0.0039 (10)	−0.0169 (11)
C11	0.0272 (12)	0.0300 (12)	0.0295 (13)	−0.0138 (10)	0.0028 (10)	−0.0132 (11)
C12	0.0363 (14)	0.0367 (14)	0.0338 (14)	−0.0184 (12)	0.0005 (11)	−0.0177 (12)
C13	0.0357 (14)	0.0429 (15)	0.0292 (14)	−0.0206 (12)	−0.0040 (11)	−0.0106 (12)
C14	0.0336 (14)	0.0344 (14)	0.0421 (16)	−0.0106 (11)	−0.0100 (12)	−0.0062 (12)
C15	0.0331 (14)	0.0287 (13)	0.0433 (16)	−0.0105 (11)	−0.0039 (11)	−0.0140 (12)
C16	0.0244 (12)	0.0277 (12)	0.0292 (13)	−0.0104 (10)	0.0013 (9)	−0.0119 (11)
C17	0.0365 (14)	0.0369 (14)	0.0331 (14)	−0.0144 (11)	0.0043 (11)	−0.0178 (12)
C18	0.0488 (17)	0.0475 (16)	0.0314 (15)	−0.0184 (14)	0.0109 (12)	−0.0201 (13)
C19	0.0448 (16)	0.0441 (16)	0.0313 (15)	−0.0176 (13)	0.0085 (12)	−0.0080 (13)
C20	0.0331 (14)	0.0315 (13)	0.0313 (14)	−0.0133 (11)	0.0015 (11)	−0.0048 (11)
C21	0.0267 (12)	0.0217 (11)	0.0319 (14)	−0.0083 (10)	−0.0021 (10)	−0.0066 (10)
C22	0.0275 (12)	0.0218 (11)	0.0289 (13)	−0.0102 (10)	−0.0018 (10)	−0.0063 (10)
C23	0.0457 (16)	0.0325 (14)	0.0395 (16)	−0.0207 (12)	−0.0018 (12)	−0.0131 (12)
C24	0.0565 (19)	0.0376 (15)	0.055 (2)	−0.0319 (14)	−0.0009 (15)	−0.0108 (14)
C25	0.0471 (17)	0.0406 (16)	0.0431 (18)	−0.0264 (14)	0.0040 (13)	−0.0039 (14)
C26	0.0293 (13)	0.0244 (12)	0.0354 (14)	−0.0091 (10)	−0.0021 (10)	−0.0147 (11)
C27	0.0287 (13)	0.0279 (12)	0.0312 (14)	−0.0078 (10)	0.0004 (10)	−0.0159 (11)
C28	0.0367 (15)	0.0400 (15)	0.0407 (16)	−0.0132 (12)	0.0038 (12)	−0.0246 (13)
C29	0.0444 (16)	0.0550 (18)	0.0351 (16)	−0.0136 (14)	0.0075 (12)	−0.0283 (14)
C30	0.0534 (18)	0.0498 (17)	0.0339 (16)	−0.0197 (14)	0.0142 (13)	−0.0166 (14)
C31	0.0435 (16)	0.0394 (15)	0.0397 (16)	−0.0214 (13)	0.0137 (12)	−0.0190 (13)
C32	0.0265 (12)	0.0276 (12)	0.0293 (13)	−0.0082 (10)	0.0029 (10)	−0.0146 (11)
C33	0.050 (2)	0.106 (3)	0.065 (3)	−0.028 (2)	−0.0050 (18)	−0.010 (2)

### Geometric parameters (Å, º)

**Table:**

Co1—O1	1.8995 (16)	C10—C11	1.416 (3)
Co1—O2	1.8932 (16)	C11—C12	1.421 (3)
Co1—N1	1.9374 (19)	C11—C16	1.421 (3)
Co1—N2	1.9142 (17)	C12—H12	0.9300
Co1—N3	1.9160 (17)	C12—C13	1.349 (4)
Co1—N4	1.9315 (19)	C13—C14	1.399 (4)
Co2—Cl3	2.2406 (9)	C14—H14	0.9300
Co2—Cl4	2.2637 (8)	C14—C15	1.370 (3)
Co2—Cl5	2.2443 (9)	C15—H15	0.9300
Co2—O3	2.026 (2)	C15—C16	1.407 (3)
Cl1—C29	1.743 (3)	C17—H17	0.9300
Cl2—C13	1.746 (2)	C17—C18	1.402 (4)
O1—C32	1.311 (3)	C18—H18	0.9300
O2—C16	1.321 (3)	C18—C19	1.356 (4)
O3—H3A	0.852 (10)	C19—H19	0.9300
O3—C33	1.417 (4)	C19—C20	1.420 (4)
N1—C1	1.331 (3)	C20—C21	1.410 (3)
N1—C5	1.371 (3)	C20—C25	1.415 (4)
N2—C9	1.414 (3)	C21—C22	1.415 (3)
N2—C10	1.302 (3)	C22—C23	1.374 (3)
N3—C22	1.425 (3)	C23—H23	0.9300
N3—C26	1.290 (3)	C23—C24	1.407 (4)
N4—C17	1.328 (3)	C24—H24	0.9300
N4—C21	1.376 (3)	C24—C25	1.367 (4)
C1—H1	0.9300	C25—H25	0.9300
C1—C2	1.398 (3)	C26—H26	0.9300
C2—H2	0.9300	C26—C27	1.425 (3)
C2—C3	1.360 (4)	C27—C28	1.423 (3)
C3—H3	0.9300	C27—C32	1.424 (3)
C3—C4	1.414 (4)	C28—H28	0.9300
C4—C5	1.405 (3)	C28—C29	1.355 (4)
C4—C6	1.411 (3)	C29—C30	1.396 (4)
C5—C9	1.409 (3)	C30—H30	0.9300
C6—H6	0.9300	C30—C31	1.370 (4)
C6—C7	1.362 (4)	C31—H31	0.9300
C7—H7	0.9300	C31—C32	1.414 (3)
C7—C8	1.395 (4)	C33—H33A	0.9600
C8—H8	0.9300	C33—H33B	0.9600
C8—C9	1.380 (3)	C33—H33C	0.9600
C10—H10	0.9300		
			
O1—Co1—N1	88.81 (8)	C16—C11—C12	119.4 (2)
O1—Co1—N2	87.06 (7)	C11—C12—H12	119.7
O1—Co1—N3	95.09 (7)	C13—C12—C11	120.7 (2)
O1—Co1—N4	178.94 (8)	C13—C12—H12	119.7
O2—Co1—O1	90.31 (7)	C12—C13—Cl2	119.68 (19)
O2—Co1—N1	179.05 (8)	C12—C13—C14	120.8 (2)
O2—Co1—N2	95.45 (7)	C14—C13—Cl2	119.5 (2)
O2—Co1—N3	87.66 (7)	C13—C14—H14	120.2
O2—Co1—N4	88.64 (8)	C15—C14—C13	119.6 (2)
N2—Co1—N1	84.13 (8)	C15—C14—H14	120.2
N2—Co1—N3	176.22 (8)	C14—C15—H15	119.0
N2—Co1—N4	93.16 (8)	C14—C15—C16	122.0 (2)
N3—Co1—N1	92.78 (8)	C16—C15—H15	119.0
N3—Co1—N4	84.75 (8)	O2—C16—C11	124.5 (2)
N4—Co1—N1	92.24 (8)	O2—C16—C15	118.0 (2)
Cl3—Co2—Cl4	111.68 (3)	C15—C16—C11	117.4 (2)
Cl3—Co2—Cl5	117.17 (3)	N4—C17—H17	118.8
Cl5—Co2—Cl4	113.63 (3)	N4—C17—C18	122.4 (2)
O3—Co2—Cl3	104.95 (7)	C18—C17—H17	118.8
O3—Co2—Cl4	106.02 (7)	C17—C18—H18	119.9
O3—Co2—Cl5	101.79 (7)	C19—C18—C17	120.2 (2)
C32—O1—Co1	125.83 (14)	C19—C18—H18	119.9
C16—O2—Co1	125.11 (14)	C18—C19—H19	120.3
Co2—O3—H3A	119.0 (19)	C18—C19—C20	119.3 (2)
C33—O3—Co2	124.2 (2)	C20—C19—H19	120.3
C33—O3—H3A	110.8 (17)	C21—C20—C19	117.4 (2)
C1—N1—Co1	129.02 (16)	C21—C20—C25	117.9 (2)
C1—N1—C5	118.4 (2)	C25—C20—C19	124.6 (2)
C5—N1—Co1	112.51 (14)	N4—C21—C20	122.1 (2)
C9—N2—Co1	113.45 (14)	N4—C21—C22	116.5 (2)
C10—N2—Co1	124.82 (16)	C20—C21—C22	121.4 (2)
C10—N2—C9	121.72 (18)	C21—C22—N3	113.31 (19)
C22—N3—Co1	112.80 (15)	C23—C22—N3	127.5 (2)
C26—N3—Co1	125.42 (16)	C23—C22—C21	119.2 (2)
C26—N3—C22	121.77 (19)	C22—C23—H23	120.2
C17—N4—Co1	129.00 (16)	C22—C23—C24	119.5 (3)
C17—N4—C21	118.4 (2)	C24—C23—H23	120.2
C21—N4—Co1	112.58 (15)	C23—C24—H24	118.9
N1—C1—H1	118.8	C25—C24—C23	122.1 (2)
N1—C1—C2	122.3 (2)	C25—C24—H24	118.9
C2—C1—H1	118.8	C20—C25—H25	120.1
C1—C2—H2	120.1	C24—C25—C20	119.8 (3)
C3—C2—C1	119.7 (2)	C24—C25—H25	120.1
C3—C2—H2	120.1	N3—C26—H26	117.2
C2—C3—H3	120.0	N3—C26—C27	125.5 (2)
C2—C3—C4	120.0 (2)	C27—C26—H26	117.2
C4—C3—H3	120.0	C28—C27—C26	117.2 (2)
C5—C4—C3	116.9 (2)	C28—C27—C32	119.4 (2)
C5—C4—C6	118.5 (2)	C32—C27—C26	123.4 (2)
C6—C4—C3	124.6 (2)	C27—C28—H28	119.6
N1—C5—C4	122.6 (2)	C29—C28—C27	120.9 (2)
N1—C5—C9	116.6 (2)	C29—C28—H28	119.6
C4—C5—C9	120.8 (2)	C28—C29—Cl1	120.5 (2)
C4—C6—H6	120.1	C28—C29—C30	120.5 (2)
C7—C6—C4	119.9 (2)	C30—C29—Cl1	119.0 (2)
C7—C6—H6	120.1	C29—C30—H30	120.0
C6—C7—H7	119.1	C31—C30—C29	119.9 (3)
C6—C7—C8	121.8 (2)	C31—C30—H30	120.0
C8—C7—H7	119.1	C30—C31—H31	119.0
C7—C8—H8	120.1	C30—C31—C32	122.1 (2)
C9—C8—C7	119.9 (2)	C32—C31—H31	119.0
C9—C8—H8	120.1	O1—C32—C27	124.6 (2)
C5—C9—N2	113.18 (18)	O1—C32—C31	118.2 (2)
C8—C9—N2	127.7 (2)	C31—C32—C27	117.2 (2)
C8—C9—C5	119.1 (2)	O3—C33—H33A	109.5
N2—C10—H10	117.3	O3—C33—H33B	109.5
N2—C10—C11	125.5 (2)	O3—C33—H33C	109.5
C11—C10—H10	117.3	H33A—C33—H33B	109.5
C10—C11—C12	116.9 (2)	H33A—C33—H33C	109.5
C10—C11—C16	123.6 (2)	H33B—C33—H33C	109.5
			
Co1—O1—C32—C27	4.7 (3)	N4—C17—C18—C19	1.1 (4)
Co1—O1—C32—C31	−176.48 (18)	N4—C21—C22—N3	−1.5 (3)
Co1—O2—C16—C11	10.9 (3)	N4—C21—C22—C23	178.0 (2)
Co1—O2—C16—C15	−171.66 (17)	C1—N1—C5—C4	0.5 (3)
Co1—N1—C1—C2	176.56 (18)	C1—N1—C5—C9	−178.9 (2)
Co1—N1—C5—C4	−177.40 (18)	C1—C2—C3—C4	1.9 (4)
Co1—N1—C5—C9	3.2 (2)	C2—C3—C4—C5	−2.2 (4)
Co1—N2—C9—C5	−1.6 (2)	C2—C3—C4—C6	177.1 (3)
Co1—N2—C9—C8	178.6 (2)	C3—C4—C5—N1	1.1 (3)
Co1—N2—C10—C11	4.4 (3)	C3—C4—C5—C9	−179.6 (2)
Co1—N3—C22—C21	3.2 (2)	C3—C4—C6—C7	−179.6 (3)
Co1—N3—C22—C23	−176.3 (2)	C4—C5—C9—N2	179.5 (2)
Co1—N3—C26—C27	3.6 (3)	C4—C5—C9—C8	−0.7 (3)
Co1—N4—C17—C18	179.5 (2)	C4—C6—C7—C8	−0.8 (4)
Co1—N4—C21—C20	179.28 (19)	C5—N1—C1—C2	−0.9 (4)
Co1—N4—C21—C22	−0.8 (3)	C5—C4—C6—C7	−0.3 (4)
Cl1—C29—C30—C31	−178.0 (2)	C6—C4—C5—N1	−178.3 (2)
Cl2—C13—C14—C15	177.9 (2)	C6—C4—C5—C9	1.0 (3)
Cl3—Co2—O3—C33	49.4 (3)	C6—C7—C8—C9	1.2 (4)
Cl4—Co2—O3—C33	−68.9 (3)	C7—C8—C9—N2	179.4 (2)
Cl5—Co2—O3—C33	172.0 (3)	C7—C8—C9—C5	−0.4 (3)
O1—Co1—O2—C16	−97.32 (18)	C9—N2—C10—C11	−176.3 (2)
O1—Co1—N1—C1	−93.7 (2)	C10—N2—C9—C5	179.0 (2)
O1—Co1—N1—C5	83.96 (15)	C10—N2—C9—C8	−0.8 (4)
O1—Co1—N2—C9	−86.47 (15)	C10—C11—C12—C13	178.9 (2)
O1—Co1—N2—C10	92.93 (19)	C10—C11—C16—O2	−1.6 (4)
O1—Co1—N3—C22	178.17 (15)	C10—C11—C16—C15	−179.1 (2)
O1—Co1—N3—C26	−0.9 (2)	C11—C12—C13—Cl2	−178.57 (19)
O2—Co1—O1—C32	−90.77 (19)	C11—C12—C13—C14	0.0 (4)
O2—Co1—N2—C9	−176.51 (15)	C12—C11—C16—O2	175.9 (2)
O2—Co1—N2—C10	2.9 (2)	C12—C11—C16—C15	−1.6 (3)
O2—Co1—N3—C22	−91.72 (16)	C12—C13—C14—C15	−0.6 (4)
O2—Co1—N3—C26	89.2 (2)	C13—C14—C15—C16	0.1 (4)
O2—Co1—N4—C17	−89.4 (2)	C14—C15—C16—O2	−176.7 (2)
O2—Co1—N4—C21	89.83 (16)	C14—C15—C16—C11	0.9 (4)
N1—Co1—O1—C32	89.60 (19)	C16—C11—C12—C13	1.1 (4)
N1—Co1—N2—C9	2.63 (15)	C17—N4—C21—C20	−1.4 (3)
N1—Co1—N2—C10	−178.0 (2)	C17—N4—C21—C22	178.6 (2)
N1—Co1—N3—C22	89.12 (16)	C17—C18—C19—C20	−1.3 (4)
N1—Co1—N3—C26	−90.0 (2)	C18—C19—C20—C21	0.3 (4)
N1—Co1—N4—C17	90.2 (2)	C18—C19—C20—C25	179.6 (3)
N1—Co1—N4—C21	−90.54 (16)	C19—C20—C21—N4	1.1 (4)
N1—C1—C2—C3	−0.2 (4)	C19—C20—C21—C22	−178.8 (2)
N1—C5—C9—N2	−1.1 (3)	C19—C20—C25—C24	−179.8 (3)
N1—C5—C9—C8	178.7 (2)	C20—C21—C22—N3	178.4 (2)
N2—Co1—O1—C32	173.79 (19)	C20—C21—C22—C23	−2.1 (4)
N2—Co1—O2—C16	−10.24 (19)	C21—N4—C17—C18	0.3 (4)
N2—Co1—N1—C1	179.2 (2)	C21—C20—C25—C24	−0.4 (4)
N2—Co1—N1—C5	−3.20 (15)	C21—C22—C23—C24	1.0 (4)
N2—Co1—N4—C17	5.9 (2)	C22—N3—C26—C27	−175.4 (2)
N2—Co1—N4—C21	−174.79 (16)	C22—C23—C24—C25	0.3 (4)
N2—C10—C11—C12	175.8 (2)	C23—C24—C25—C20	−0.6 (5)
N2—C10—C11—C16	−6.6 (4)	C25—C20—C21—N4	−178.3 (2)
N3—Co1—O1—C32	−3.09 (19)	C25—C20—C21—C22	1.8 (4)
N3—Co1—O2—C16	167.61 (18)	C26—N3—C22—C21	−177.7 (2)
N3—Co1—N1—C1	1.4 (2)	C26—N3—C22—C23	2.8 (4)
N3—Co1—N1—C5	179.00 (16)	C26—C27—C28—C29	−177.3 (2)
N3—Co1—N4—C17	−177.2 (2)	C26—C27—C32—O1	−1.9 (4)
N3—Co1—N4—C21	2.05 (16)	C26—C27—C32—C31	179.3 (2)
N3—C22—C23—C24	−179.5 (2)	C27—C28—C29—Cl1	177.1 (2)
N3—C26—C27—C28	175.6 (2)	C27—C28—C29—C30	−2.3 (4)
N3—C26—C27—C32	−2.6 (4)	C28—C27—C32—O1	179.9 (2)
N4—Co1—O2—C16	82.80 (18)	C28—C27—C32—C31	1.1 (3)
N4—Co1—N1—C1	86.2 (2)	C28—C29—C30—C31	1.4 (5)
N4—Co1—N1—C5	−96.15 (16)	C29—C30—C31—C32	0.8 (5)
N4—Co1—N2—C9	94.56 (15)	C30—C31—C32—O1	179.1 (2)
N4—Co1—N2—C10	−86.04 (19)	C30—C31—C32—C27	−2.0 (4)
N4—Co1—N3—C22	−2.88 (15)	C32—C27—C28—C29	1.1 (4)
N4—Co1—N3—C26	178.0 (2)		

### Hydrogen-bond geometry (Å, º)

**Table:**

*D*—H···*A*	*D*—H	H···*A*	*D*···*A*	*D*—H···*A*
O3—H3*A*···Cl4^i^	0.85 (2)	2.25 (2)	3.081 (3)	166 (2)
C1—H1···Cl3^ii^	0.93	2.72	3.532 (3)	146
C10—H10···O1^iii^	0.93	2.47	3.324 (3)	152
C33—H33*A*···Cl5^i^	0.96	2.82	3.745 (4)	161

Symmetry codes: (i) −*x*+1, −*y*+2, −*z*; (ii) −*x*+1, −*y*+1, −*z*+1; (iii) −*x*, −*y*, −*z*+1.
